# College Note

**Published:** 1903-10

**Authors:** 


### COLLEGE NOTE.

The directors of the Memphis Medical College have established a lectureship, named in memory of the late William E. Rogers, M. D., founder of that institution.

The lectureship was established upon the suggestion of the faculty. Once during each session a lecture will be given by a specialist in some branch of medical science, at the invitation of the faculty.

The first lecture will be given about the middle of November, 1903, by Dr. Charles H. Hughes, of Saint Louis. Dr. Hughes is a professor in the medical department of Washington University.

